# 

**Published:** 1895-06

**Authors:** 


### CONTENTS.

COMMUNICATIONS.

Riggs' Disease; or, "What you Will" ........ 261

PROCEEDINGS.

Twenty Sixth Annual Session of the Alabama Dental Association, 1895 ........ 294

COMMENCEMENTS.

University of Buffalo—Dental Department ........ 307

SELECTIONS.

Second Childhood ........ 293
Faith Cure ........ 293
Statistics ........ 293
His Tooth Exploded ........ 306
Health Commandments ........ 307
The President of the American Medical Association ........ 308

NOTICES.

National Association of Dental Examiners ........ 309
The American Dental Association ........ 309
East Tennessee Dental Association ........ 310
Illinois State Dental Society ........ 310

EDITORIAL.

A Morning Star ........ 311
National Association of Dental Faculties ........ 311
Tri-State Meeting ........ 312

